# An unexpected case of a dog from Poland co-infected with *Dirofilaria repens* and *Dirofilaria Immitis*

**DOI:** 10.1186/s12917-024-03921-3

**Published:** 2024-02-23

**Authors:** Mateusz Pękacz, Katarzyna Basałaj, Martina Miterpáková, Zbigniew Rusiecki, Diana Stopka, Dominika Graczyk, Anna Zawistowska-Deniziak

**Affiliations:** 1 Division of Parasitology, Department of Preclinical Sciences, Faculty of Veterinary Medicine, Warsaw University of Life Sciences-SGGW, Ciszewskiego 8, Warsaw, 02-776 Poland; 2 Museum and Institute of Zoology, Polish Academy of Sciences, Wilcza 64, Warsaw, 00-679 Poland; 3 Institute of Parasitology, Slovak Academy of Sciences, Hlinkova 3, Košice, 040 01 Slovakia; 4 Centrum Zdrowia Zwierząt, Brata Alberta 34, Warsaw, 05-075 Poland; 5 Division of Pathology, Department of Pathology and Veterinary Diagnostics, Faculty of Veterinary Medicine, Warsaw University of Life Sciences-SGGW, Ciszewskiego 8, Warsaw, 02-776 Poland; 6 Department of Immunology, Institute of Functional Biology and Ecology, Faculty of Biology, University of Warsaw, Miecznikowa 1, Warsaw, 02-096 Poland

**Keywords:** Dirofilariasis, Co-infection, Diagnostics, Real-time PCR

## Abstract

**Background:**

Dirofilariasis is a vector-borne disease caused by parasitic nematodes of the genus *Dirofilaria spp.*, considered an emerging concern in both veterinary and human medicine. Climate changes and human activities, such as pet travel, contribute to the spread of diseases to new non-endemic regions. Poland is dominated by subcutaneous dirofilariasis caused by *D. repens* infections. Cardiopulmonary dirofilariasis, also known as a heartworm disease is much more rare with only single autochthonous cases reported so far. Also, imported infections are observed sporadically in dogs traveling to endemic countries. In this study, we report the first case of a dog in Poland, never having traveled abroad, co-infected with *Dirofilaria repens* and *Dirofilaria immitis*.

**Case presentation:**

A 14-year-old mixed breed, an intact male dog with fever, lightly pale mucosal membranes, moderate abdominal pain, and a mild cough was presented in a veterinary clinic in Warsaw, Poland. The examination of the blood sample collected for complete morphology and biochemistry revealed the presence of live microfilariae. Presence of the DNA of both microfilariae species was detected using Real-Time PCR with species-specific primers.

**Conclusions:**

Since the remaining diagnostic methods like Knott’s test, antigen test or echocardiography did not reveal the presence of *D. immitis*, we discussed the impact of microfilariae periodicity and low worm burden infections on the limited efficiency of these techniques. We strongly recommend using a mixed diagnostic approach for the most sensitive and specific diagnosis since the ideal diagnostic method does not exist, and several factors may contribute to misdiagnosis. Furthermore, we considered factors that contribute to the uncontrolled spread of dirofilariasis such as climate changes, introduction of new species of mosquitoes competent for the transmission of the disease, and wildlife animals as an important reservoir of this parasitosis. Given that Poland shares borders with countries classified as endemic and pre-endemic for *D. immitis*, such as Slovakia and Ukraine, it is reasonable to anticipate a rise in autochthonous heartworm infections and shifts in the epidemiological pattern of dirofilariasis in the coming years.

## Background

Dirofilariasis is a vector-borne disease caused by parasites from the genus *Dirofilaria spp. Dirofilaria repens* is a causative agent of subcutaneous dirofilariasis whereas *D. immitis* causes cardiopulmonary dirofilariasis, also known as heartworm disease [1]. While the primary hosts are carnivores, both parasites exhibit zoonotic potential and can infect humans. The disease is spreading worldwide, gradually expanding into new non-endemic areas year after year. Direct causes of spreading the disease are climate changes, which lead to the introduction of new species of mosquitoes competent for disease transmission. Additionally, increased movement of pets, especially those lacking sufficient protection, contributes to the spread [2].

Both *Dirofilaria* species are widespread in Europe, but their distribution in specific regions differs. *D. repens* infections are prevalent across Europe and endemic in many countries of both Southern and Central Europe. Moreover, the risk of new endemic regions emerges in North Europe and Baltic countries, where infections have occurred more frequently in recent years [3, 4]. Heartworm infections have predominantly been observed in Southern Europe, particularly in the hyper-endemic Mediterranean region. In the rest of Europe, especially in the central part, infections have been reported only occasionally. However, according to Morchón et al. [5], the epidemiological situation is constantly changing. Over the last 10 years, the prevalence of the disease not only increased in previously endemic areas but also spread across new non-endemic regions. Single imported cases or occurrences of infected mosquitoes were reported even in Northern Europe countries like Norway [6] and Denmark [2].

Near Poland, Slovakia is a region that can be considered endemic. Over the years, *D. immitis* infections have become more and more frequent in the regions that up to this time were dominated by *D. repens* [7, 8]. Currently, heartworm disease, both in mono-infection and co-infection with *D. repens*, represents 45% of all dirofilarial infections in Slovakia. Between 2017 and 2021 mixed infections of *D. repens* and *D. immitis* represented 22.5% of all diagnosed cases of canine dirofilariasis in endemic regions of Slovakia [8]. Moreover, in 2022 an autochthonous case of human heartworm infection was confirmed in this area [9]. Interestingly, in the bordering Czech Republic, only imported cases have been identified so far [10]. A significant number of *D. immitis* infections have been detected in Germany in recent years, all of which were associated with imported or traveling dogs. In addition, in some regions of the country *D. immitis* DNA was identified in a pool of mosquitoes competent for transmission of the heartworm disease [11]. Based on these reports, Germany may be considered a pre-endemic area [5].

Beyond Poland’s eastern border, in Lithuania, a single imported case of canine cardiopulmonary dirofilariasis was identified [12] and in Belarus, *D. immitis* DNA was detected in a pool of competent mosquitoes [13]. In the case of Ukraine, data on prevalence in dogs are limited, but 1465 cases of human infections caused by *D. repens* were reported in the years 1997–2012 [14] and in the other study among 102 cases, a few were identified as *D. immitis* [15]. Based on these findings Ukraine is considered endemic for both species [16].

Poland is dominated by subcutaneous dirofilariasis. The first case of *D. repens* infection in Poland was reported in humans in 2007 [17, 18], but the patient traveled to Greece a few years earlier so invasion might have been imported. The first autochthonous infection in humans was reported in 2010 [19], with subsequent cases described in the following years [19–23]. The first case of canine dirofilariasis was described in 2009 [24, 25], and since then the prevalence of infected dogs has been increasing steadily. In 2014, the overall prevalence of infected dogs across all 16 provinces reached almost 16% [26], whereas in 2016 only in the Mazovia district the percentage of infected dogs was 38.3% [27]. In a recent study conducted in 2017–2019, the prevalence in Poland was 12% [3].

The first case of a dog infected with *D. immitis* was described in 2012 in Gdynia [28] based on the SNAP test (IDEXX) detecting adult female antigens and has been suspected to be autochthonous. Despite this, only one additional autochthonous case was described in Silesia in 2014 [29]. Importantly, physicians sporadically observe imported infections in dogs that have traveled to endemic countries (data unpublished, based on personal communication). In light of climate changes and Poland’s proximity to countries where *D. immitis* is endemic (Slovakia, Ukraine) and pre-endemic (Germany), we have every reason to expect an increase in autochthonous infections shortly.

Here we report a case of a dog from Poland co-infected with *Dirofilaria repens* and *D. immitis*, that has never traveled abroad. To our best knowledge, this is the first confirmed case of autochthonous *D. immitis* infection in a dog from Poland using molecular methods.

## Case presentation

### Case report

In autumn 2022, a 14-year-old mixed breed, an intact male dog was brought to a veterinary clinic in Warsaw, Poland, for an evaluation due to general lameness. Clinical signs included: fever (39.5 ℃), lightly pale mucosal membranes, moderate abdominal pain, and mild cough. No lymphadenopathy of the peripheral lymph nodes and no deviations from the standard image in the ultrasound examination of the abdominal cavity were observed. A blood sample was collected to complete morphology and biochemistry tests, and then anti-inflammatory and antiemetic drugs were implemented. The results are showed in the Table 1.

Because of lowered PLT the CaniV-4 rapid test was performed (One-step Canine Heartworm Antigen and *Ehrlichia canis*; *Borrelia burgdorferi*; *Anaplasma phagocytophilum* Antibody Test, Vetexpert). The test was positive on *Anaplasma* antibodies and antibiotic treatment with doxycycline at a dose of 10 mg/per kilo/day, for 7 days was implemented. After a week of treatment, the dog’s health status did not show any noticeable improvement. A follow-up blood sample was obtained for a control morphology blood test and blood smear. While no *Anaplasma* was observed in the smear, live microfilariae were detected in the native blood sample. Molecular analyzes confirmed the presence of microfilariae from both *D. repens* and *D. immitis*, with a higher quantity of *D. repens* indicated Ct (cycle threshold) values obtained through Real-Time PCR. The dog had no lumps in the skin and the control echocardiography showed no visible adult forms of *Dirofilaria spp*. in the heart. The control radiography of the thorax in dorsoventral and lateral projections showed no deviations. Based on information from the owner, the dog has never traveled abroad but it lived in the vicinity of dogs traveling outside Poland’s borders.

The dog was treated with a combination of imidacloprid 250 mg and moxidectin 62.5 mg in spot-on drops (Advocate, Bayer) according to the following treatment schedule: four doses of the drug administrated at four-week intervals. Additionally, doxycycline (10 mg/kg/day) was continued for a total of one month due to *Anaplasma sp.* and *Wolbachia.* After 2 months the follow-up blood test showed no microfilariae in the bloodstream and all the blood and biochemistry parameters were in the correct ranges. During the following months, the dog was under constant medical care.

### Knott’s test

**Fig. 1:**
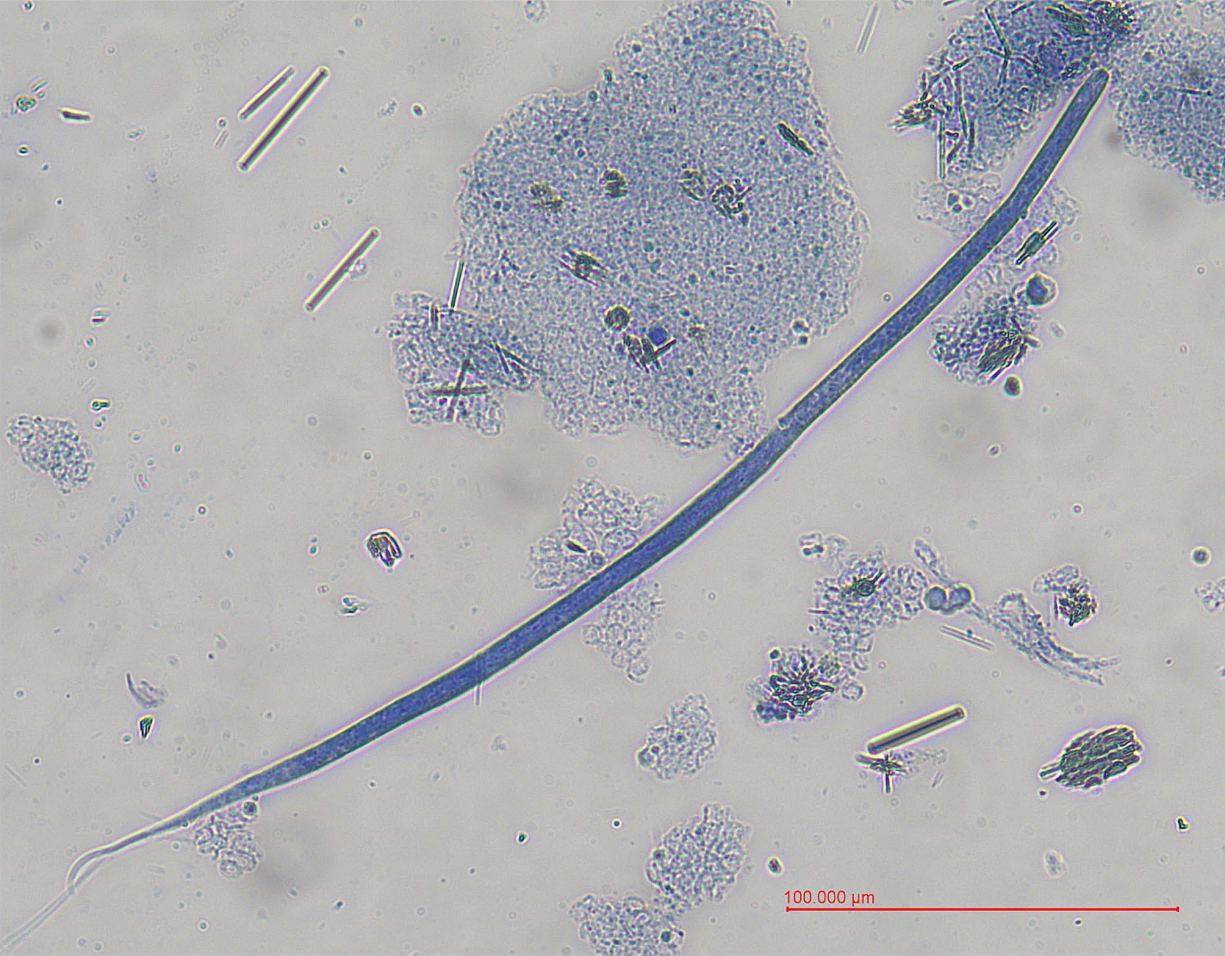
Microfilaria of *Dirofilaria repens* identified in Knott’s test during examination of a dog’s blood sample observed under 40× magnification

One ml of blood was mixed with 9 ml of 4% formalin and centrifuged for 10 min at 500 × g. The supernatant was discarded and the pellet was stained with 1% methylene blue. One drop was deposited on the glass slide and examined under the light microscope at 10× and 40× magnification. The Knott’s test revealed the presence of microfilariae, displaying features typical of *D. repens*, including a blunt-end head with a short cephalic space, a distinct pair of nuclei, and a hooked tail. The average length and width of the presented microfilariae were measured at 364,3 μm and 6,2 μm, respectively (Fig. 1). These distinctive characteristics collectively confirm the identification of the microfilariae as belonging to *D. repens* [30, 31]. No microfilariae of *D. immitis* were detected. However, given the low intensity of microfilaremia (200 mf/ml of whole blood) and the observation of only single microfilariae on the slide, it is possible that they were omitted during the examination.There was no opportunity to repeat the examination of microfilariae as the next blood samples were collected already at a check-up visit one month after the treatment has been implemented.

### Molecular detection

300 µl of blood was used for the isolation of genomic DNA using Blood Mini Kit (AA Biotechnology) according to the manufacturer’s protocol. The isolated gDNA was used as a template in Real-Time PCR reaction with primers targeted at the *D. repens* 16s *rRNA* gene described in our previous study [32] and newly designed species-specific primers for *D. immitis* (ForDI 5’ ACTGATGTTATTATTCTATGTGTTTGGG 3’; RevDI 5’ TTCAAAGAATCCCACTCTAAAAACCTC 3’). The novel primers targeted the small fragment of mtDNA located between *tRNA* and *ND6* genes (starts at 3960 bp and ends at position 4109 bp), were designed based on the reference mitochondrial genomes of *D. immitis* (NC_005305.1) and *D. repens* (NC_029975.1) so that 3’ mismatches would discriminate and would not amplify *D. repens* DNA fragments.

Reactions were performed in triplicate according to the two-step fast cycling protocol (PowerUp™ SYBR™ Green Master Mix, Applied Biosystems™), followed by the melt curve step in a QuantStudio 6 Real-Time PCR system (Applied Biosystems) according to the manufacturer’s protocol. After the UDG (Uracil-DNA Glycosylase) activation step (2 min at 50 °C) followed by initial denaturation (2 min, 95 °C), 40 cycles of amplification were performed (3 s at 95 °C, 30 s at 60 °C). The reaction volume was 10 µl and consisted of 5 µl of 2× Master mix, 3 µl of the isolated DNA, 1 µl of each primer in a final concentration of 0.6 µM. Data were collected during the annealing/extension step.

The specificity of the amplified products was confirmed based on the melt curve peaks compared to the reference samples and Sanger’s sequencing. Reference samples were as follows: gDNA isolated from adult *D. repens* (*N* = 1) and *D. immitis* worm (*N* = 1); gDNA isolated from the blood of dogs infected with *D. repens* (*N* = 10) and *D. immitis* (*N* = 10); gDNA isolated from the blood of dogs co-infected with both species (*N* = 5). All samples related to *D. immitis* were derived from Slovakia.

**Fig. 2:**
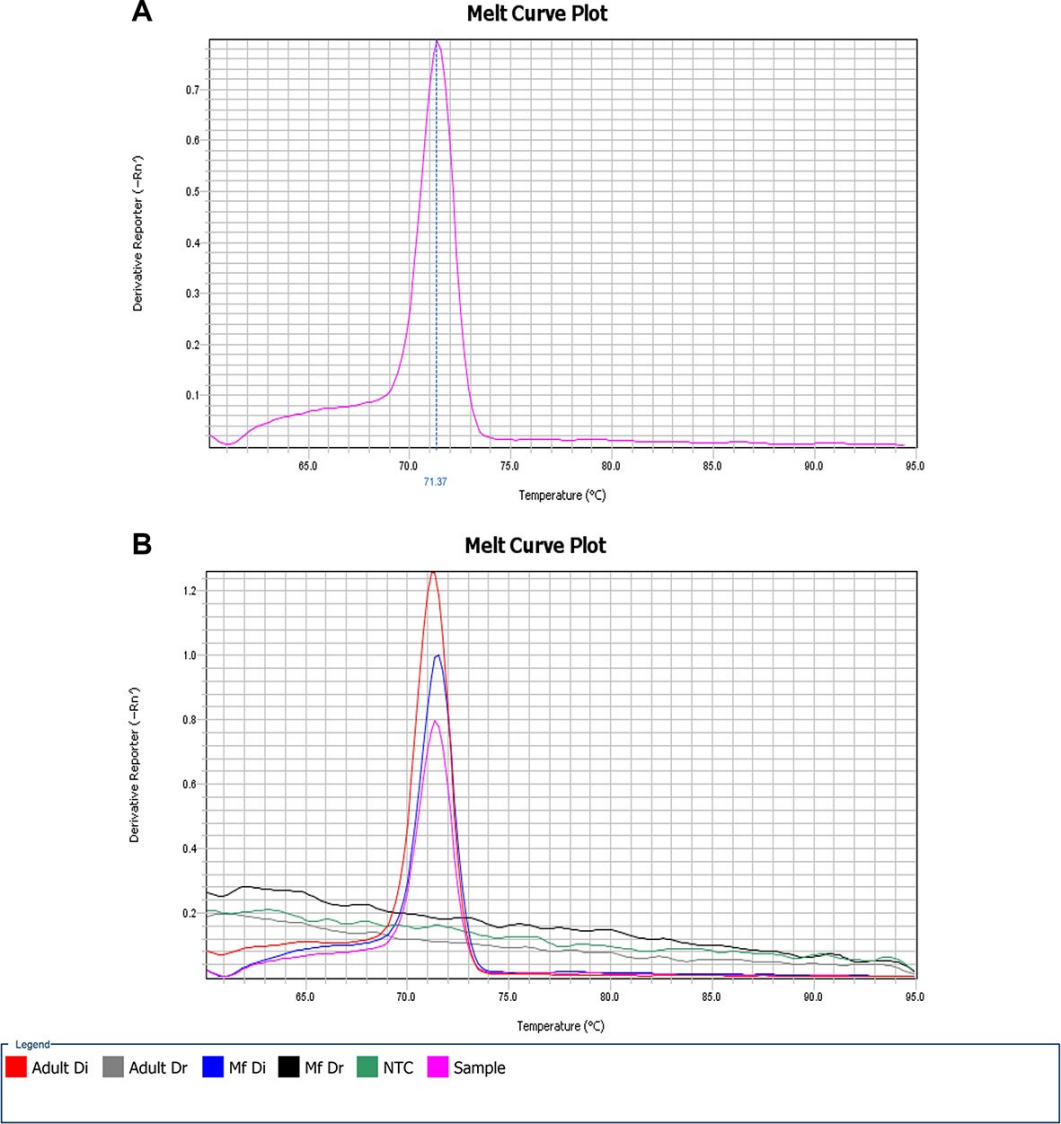
Melt curve analysis of products amplified using gene-specific primers for *D. immitis*: (**A**) reveals a distinct peak with a specific melting temperature (Tm) corresponding to the amplicon obtained from the tested sample; (**B**) demonstrates primer specificity assessed with genomic DNA isolated from adult *D. immitis* (Adult Di) and *D. repens* (Adult Dr) worms, blood from dogs infected with *D. immitis* (Mf Di), and *D. repens* (Mf Dr). NTC implies “no target control”

The specificity of the designed primers was confirmed in all reference samples. Melt curve analysis revealed peaks specific for *D. immitis* and *D. repens* with Tm ∼ 71 °C (Fig. 2) and ∼ 72 °C, respectively. The novel primers specific for *D. immitis* amplified only *D. immitis* gDNA (microfilariae or adult). No amplification was observed in reactions with *D. repens* gDNA (microfilariae or adult).

Both genes of interest were amplified with the gDNA isolated from the blood of the described dog which indicates co-infection. Tm of melt curves were consistent with reference samples and sequencing of the amplified DNA fragments showed almost 100% similarity to *s16* rDNA and “DI fragment” for *D. repens* and *D. immitis*, respectively. The sequencing of the amplicons was outsourced to Genomed S.A., where each sample was bidirectionally sequenced using the same gene-specific primers employed in our real-time PCR analysis.

Interestingly, the product specific for *D. immitis* presented a much higher Ct value (Ct ∼ 35), than the one specific for *D. repens* (Ct ∼ 29). That indicates the predominance of *D. repens* in the bloodstream and seems to correspond to the Knott’s test result, where *D. immitis* microfilariae have not been even detected. We assume that the number of *D. immitis* microfilariae was too low to be noticeable in Knott’s test. There may be a few explanations for this phenomenon related to the biology of the parasite.

## Discussion and conclusions

Mixed infections of both species are not uncommon and have already been reported in several countries [10, 33–35] with a relatively high prevalence. For example, in Romania’s 2015 report, mixed infections represented 23.91% of all positive samples [36]. Interestingly, the predominance of microfilariae of one of the species over the other has also been frequently observed, e.g. 7780 mf/ml and 427 mf/ml of *D. immitis* and *D. repens*, respectively [34, 37]. A similar scenario has been observed in naturally co-infected dogs in Slovakia (personal communication, prof. Martina Miterpáková). Genchi et al. [38] observed this phenomenon in experimentally infected dogs and suggested that the interaction of both species may disrupt the progress of each other and pointed out that it may impact the further distribution of dirofilariasis in different regions. Periodic fluctuations in microfilariae levels, influenced by factors like host behavior and environmental conditions, can impact the effectiveness of standard morphological/molecular examinations. Studies suggest a link between microfilaremia dynamics and host habits, as well as vector activity in specific regions [37]. Seasonal variation in microfilaremia is evident, peaking during summer [39–41]. Although limited, research on mixed infections in dogs reveals a shared circadian rhythm between parasites, with peripheral microfilaremia highest at 1 am and lowest between 5 and 8 am. Intriguingly some cases showed zero microfilariae of one species between 9 and 11 am [37], supporting the assumption that during low-count phases, microfilariae concentrate in lung vessels [42].

These findings appear consistent with our situation, as the blood sample for differentiating the infection collected at 10.30 am corresponds to a period of low peripheral microfilaremia (200 mf/ml), possibly contributing to the absence of *D. immitis* in the Knott’s test. However, we recognize that sampling at a single time point is a limitation and may not definitively conclude the absence of *D. immitis* due to time of day. Regrettably, additional sampling at varied time points was not feasible in this case.

In addition, a negative result of the antigen test and no sign of adult worms may indicate low worm burden infection [43], which also complies with AHS (American Heartworm Society) [44] and ESDA (European Society of Dirofilariosis and Angiostrongylosis) [45] directives. While echocardiography is crucial for assessing the severity of the infection, it may also be misleading, particularly in lightly infected dogs, where worms could be beyond the field of view. Sporadically, *D. immitis* worms have also been reported in various atypical locations [46, 47]. As described, the patient did not show any specific heartworm symptoms, only occasional coughing and general lameness were observed. The infection should be considered “mild” and “Class 1 with low risk of thromboembolic complications”, according to AHS and ESDA directives, respectively [44, 45].

Here, we report the first case of a naturally co-infected dog with both *D. repens* and *D. immitis* in Poland, marking the third documented case of heartworm infection in the country overall. While the country is not considered pre-endemic, we believe heartworm disease may be underestimated, leading to potential undiagnosed or misdiagnosed cases.

Climate changes introduce vectors competent for transmission and extend the exposure time to infection, allowing more *Dirofilaria* generations in a season. Mosquito larval development, influenced by temperature, shows the fastest progress at 28–30 °C, taking 8–9 days for *D. immitis* and 9–13 for *D. repens*, with a threshold of 14 °C below which *Dirofilaria* will not evolve [48]. This information led to the creation of a seasonal heartworm (HW) transmission model, enabling the prediction of *Dirofilaria* occurrences [49–51]. Recently, suitable conditions were occasionally observed in Northern European countries such as Sweden, Norway, Finland, and Denmark [2]. Human activities are also crucial for the transmission of the disease to non-endemic regions. Traveling with insufficiently protected and/or not properly examined pets contribute to the appearance of new outbreaks of the disease, that within a short period may lead to the endemization in new areas. According to Fuehrer et al. [2], more than 30% of dogs in Poland are kept outside overnight, placing them at an increased risk of mosquito bites.

Although the awareness of dirofilariasis increased in recent years and epidemiological data is being updated locally, still there are some research areas where the data is limited. One of them is molecular xenomonitoring which recently was improved in many European countries and several protocols have been described [52–54]. In Poland, only two studies have been conducted in this field, [55, 56] and provided estimates of the infection rate (EIR) at 1.57% for *D. repens* in the Central part of Poland. Neither *D. immitis*, nor *D. repens* DNA was detected in vectors collected from Southern West part of Poland. Although examining hundreds or even thousands of mosquitoes for a single infected individual might seem economically questionable, xenomonitoring offers valuable insights into transmission risk and the actual epidemiological status.

Unfortunately, the ongoing neglect of infections in free-living carnivores remains a significant contributing factor to the uncontrolled spread of dirofilariasis. Foxes, jackals, wolves, and raccoon dogs in Europe have been identified with infections from both *Dirofilaria* species. Recent findings in beech martens [57] and European badgers [58] suggest a potentially broader natural host range, prompting discussions on their roles as reservoir hosts. In a recent study, Alsarraf et al. [59] reported that the overall prevalence in Poland reached 3.13%, which corresponds with similar studies conducted in other European countries [60–62]. Interestingly, in neighboring Slovakia, *D. repens* infections were detected in 54.97–57.4% of examined foxes [63, 64]. Moreover, only in the Tatry region (the natural borderline between Poland and Slovakia), the prevalence in foxes reached 24.6% [65]. In light of possible patent infections and the absence of preventive or therapeutic interventions in fox populations, these animals could facilitate access to microfilariae for new mosquito genera, particularly given their nocturnal habits and proximity to human habitats. Foxes’ nomadic tendencies and capacity for long-distance travel further amplify the risk of disease spread. Nonetheless, the infrequent occurrence of microfilaremia and the inconsistency in available data mean that the true impact of foxes as reservoirs is still subject to debate.

As Poland is surrounded by at least two endemic (Slovakia, Ukraine) and one pre-endemic (Germany) country, we suppose that subsequent cases of both imported and autochthonous infections will be reported more frequently in the following years. Interestingly, in a recent study, Alsarraf et al. demonstrated that genetic diversity among populations of dogs infected with both *D. repens* and *D. immitis* appears to be linked to their geographical origin [16, 66]. The ongoing cultivation of this field of study could significantly contribute to understanding the origin of infections and monitoring the potential migration between populations.

In summary, following the OneHealth approach, it is essential to rigorously monitor the epidemiological situation not just in dogs but also in humans, wildlife animals, and insects. Our case supports the thesis that a mixed diagnostic approach based on morphological, molecular, and serological techniques provides the most sensitive and specific diagnosis.

**Table 1:** Hematological and biochemical parameters of the investigated dog at the time of the initial presentation in the veterinary clinic. Bold font is used to indicate increased or lowered parameters

	Results	Unit	Reference intervals
**Morphology**			
Leukocytes	11.10	G/l	6.00–12.00
Erythrocytes	**5.23**	T/l	5.50–8.00
Hemoglobin	7.70	mmol/l	7.45–11.17
Hematocrit	0.37	l/l	0.37–0.55
MCV	71	Fl	60–77
MCH	1.47	Fmol	1.18–1.49
MCHC	20.8	mmol/l	19.8–22.3
RDW	14	%	13–19
Platelets	**41**	G/I	200–580
**Manual Blood Smear**			
Banded neutrophils	3	%	0–3
Segmented neutrophils	**92**	%	60–77
Lymphocytes	**5**	%	12–30
**Biochemistry**			
AST (aspartate aminotransferase)	**54.0**	U/l	3.0–45.0
ALT (alanine aminotransferase	**280.0**	U/l	5.0–60.0
AP (alkaline phosphatase)	**1415.0**	U/l	5.0–155.0
Glucose	106.0	mg/dl	70.0–120.0
Creatinine	**0.7**	mg/dl	0.8–1.7
Urea	**18.0**	mg/dl	20.0–50.0
Total protein	74.0	g/l	55.0–75.0
Bilirubin	0.4	mg/ml	0.2–0.9
Albumins	38.0	g/l	29.0–43.0
GGT (gamma-glutamyl transferase)	**33.0**	U/l	5.0–25.0
Calcium	10.0	mg/ml	8.4–11.5
Phosphorus	2.7	mg/ml	2.5–6.3
Magnesium	1.8	mg/ml	1.7–2.9
Total cholesterol	239.0	mg/ml	128.0–360.0
LDH (lactate dehydrogenase)	149.0	U/l	80.0–1683.0
CK (creatine kinase)	78.0	U/l	5.0–467.0
Triglycerides	72.0	mg/dl	18.0–115.0
Sodium	151.8	mmol/l	139.1–156.5
Potassium	4.8	mmol/l	4.1–5.4

## Data Availability

Derived data supporting the findings of this study are available from the corresponding author MP on request.
